# Differentiation Governance of Rural Human Settlement Environments in China: Knowledge Mapping and Visualization

**DOI:** 10.3390/ijerph20054209

**Published:** 2023-02-27

**Authors:** Xin Dai, Junying Zhang, Xuehang Sun, Junjie Li, Bangfan Liu

**Affiliations:** 1 Institute of Marxism Chinese Academy of Social Sciences, Beijing 100732, China; 2 School of Public Administration, Yanshan University, Qinhuangdao 066004, China; 3 Hebei Public Policy Evaluation and Research Center, Qinhuangdao 066004, China

**Keywords:** China, rural human settlement environment, differentiation governance, living environment

## Abstract

To further promote the effective governance of rural human settlements in China, it is necessary to summarize and organize the research on rural human settlements that has been undertaken in the last decade. This paper analyzes the current status of rural human settlements research from the perspectives of Chinese literature and English literature. It takes the core documents included in WOS (Web of Science) and CNKI (Chinese National Knowledge Infrastructure) as samples, and produces a visual analysis of the authors, institutions, disciplines, and research hotspots for rural human settlements research with the help of CiteSpace V and other measurement software, focus on identifying the similarities and differences between CNKI and WOS in the study of rural human settlements. The results show that the number of papers is increasing; cooperation between Chinese researchers and institutions needs to be further strengthened; the existing research has achieved interdisciplinary integration; the research hotspots are converging, but China pays more attention to the study of the hard environment, such as the macro level of rural human settlements and the natural ecological environments of residence, and lacks insight into the soft environment, such as the main body of residences, social relations, and individual needs in the urban fringe. This study is conducive to promoting the integrated development of urban and rural areas in China, promoting the revitalization and development of rural areas in China, and achieving social equity.

## 1. Introduction

The rural human settlement environment refers to the places that rural residents rely on for production, life, and residence. With the implementation of the rural revitalization strategy, rural human settlements have attracted much attention and become an important part of rural revitalization. The report of the 19th National Congress of the Communist Party of China officially proposed action to be carried out for the rectification of rural human settlement environments. Since then, the study of rural human settlement environments has gained the attention of domestic scholars. In February 2018, the General Offices of the CPC Central Committee and the State Council issued the Three-year Action Plan for the Improvement of Rural Human Settlements (2018–2020). In 2021, the General Offices of the CPC Central Committee and the State Council issued the five-year Action Plan for the improvement of Rural Human Settlements (2021–2025). The improvement of rural human settlements has entered a new stage [1]. Through improvements made in recent years in garbage treatment, sewage treatment, sanitation and hygiene, public services, village appearance, and other areas, China rural human settlements have achieved a great deal; at the same time, however, certain problems remain, and we must continue to learn, explore, and improve in governance [2]. Compared with the short exploration time in China, international researchers began to look into rural residential environments earlier. Therefore, there are many research studies that Chinese scholars can learn from, so as to improve the construction of the rural residential environment. Chinese scholars’ research into rural human settlement environments is mostly qualitative research; most of these studies are focused on the study of governance issues and countermeasures in human settlement environments, the evolution of the context, and other specific themes. Moreover, there are few reviews and summaries of the English literature, and even fewer studies combining Chinese and English literatures, leading to Chinese scholars’ failure to actively learn from and absorb the results of international research. Therefore, it is necessary to conduct an analysis of the literature on rural human settlements in the past decade. In this study, we focus on the WOS (Web of Science) and CNKI (Chinese National Knowledge Infrastructure) databases, and we carry out visual analysis with the help of literature measurement software. How do we understand the current situation of international research, and mainly explore what is the current focus of rural human settlements research? What is the research trend and direction? What are the similarities and differences between China and other countries? We seek to summarize international research achievements, learn from international excellent achievements, make China’s rural human settlements research connect with the world, further promote the construction and development of China’s rural human settlements, and realize rural revitalization.

## 2. Data Sources and Research Tools

In order to fully reflect the reliability and comprehensiveness of the data, the Chinese and English documents in this paper are from the CNKI platform and WOS platform, respectively, which represent one of the most authoritative databases in Chinese and English.

### 2.1. Data Collection

Chinese studies were screened through the CNKI platform, and data sources were selected from Peking University Core, EI, and CSSCI. The search conditions were as follows: the subject was rural human settlements OR village human settlements; the document type is academic journal; the language is Chinese; the matching method was selected as accurate; the time span is 1 January 2013–8 June 2022. Due to the requirements of the research tools, the title information was exported in the form of Refworks, EndNote, and NoteExpress text, and imported into NoteExpress software for data cleaning and manual cleaning. In order to prevent the contents of master’s and doctoral theses and conferences from duplicating the contents of journals, some documents, such as master’s and doctoral theses and conference theses, as well as book reviews, conferences, solicitations, news articles, etc., which were not relevant to the research topic, were eliminated, and 292 effective documents were finally obtained. For English literature screening through WOS, the following search conditions were entered for the basic search: the database is a core collection of Web of Science; the theme is rural residential environment or rural living environment; the document types are article and review; the language is English; the time span is 1 January 2013–8 June 2022. A total of 2991 documents were searched. After the duplications were processed in the same way as that described for the Chinese documents, a total of 2990 valid documents were retained.

### 2.2. Research Tools

The research tools used were SATI4.0 (http://www.sationline.cn/#!/, Tianjin, China), Note Express (Beijing Aegean Music Technology Co., Ltd., Beijing, China), and CiteSpace V (https://citespace.podia.com/, Dalian, China), a literature measurement software used around the world. SATI is a document catalog information analysis tool, which converts text data such as CNKI, WANFANG DATA, and the China Science and Technology Journal Database, and extracts and counts data including keywords, authors, and institutions for the construction of a common word matrix and the conversion of different matrices [3]. Note Express is a professional literature retrieval and management system; its main functions include the collection of titles, title management, and other title information analysis functions. In this paper, it was mainly used to analyze the distribution of keywords, authors, and institutions. CiteSpace V is a visualization analysis software for large quantities of literature; its development was based on JAVA, which can realize the operation of literature co-citation and coupling analysis. Through the visualization analysis of literature, a map of scientific knowledge can be drawn, which can more accurately grasp the research field, understand research progression, and identify the research hot spots. For both domestic and foreign literature, this paper mainly uses this software to analyze keyword co-occurrence clustering and keyword frontier trends to achieve an overall understanding of the research topic.

Using the above research tools to systematically sort out the literature, we can clearly see the current situation and future trend in rural human settlements research, which is conducive to achieving clearer quantitative analysis and making the quantitative results and article conclusions more convincing. However, this research method also has certain limitations. Although the basic data supporting the research come from the authoritative database, it is difficult to guarantee the complete reproduction of the academic research process; in addition, data collection and analysis are divided by time period, and the latest research results cannot be entered at any time, which also creates a shortage in real time to some extent.

## 3. The Research Status and Differentiation Analysis of Rural Human Settlement Environments in CNKI

In recent years, with the acceleration of the rural revitalization strategy, rural human settlements have become an important basis for rural revitalization. To date, the governance of rural human settlement environments has attracted the attention of many scholars. The academic community has carried out extensive research on the problem of rural human settlement environments and achieved fruitful results. Subjects including the policy response to the governance of rural human settlements and the construction of the institutional system, as well as the development trajectory, have been studied and elucidated. This paper first presents a knowledge graph analysis of the CNKI research situation, so as to understand some directions and themes of the research on rural human settlements in China.

### 3.1. The Number of Published Documents of Rural Human Settlements Research

**Figure 1:**
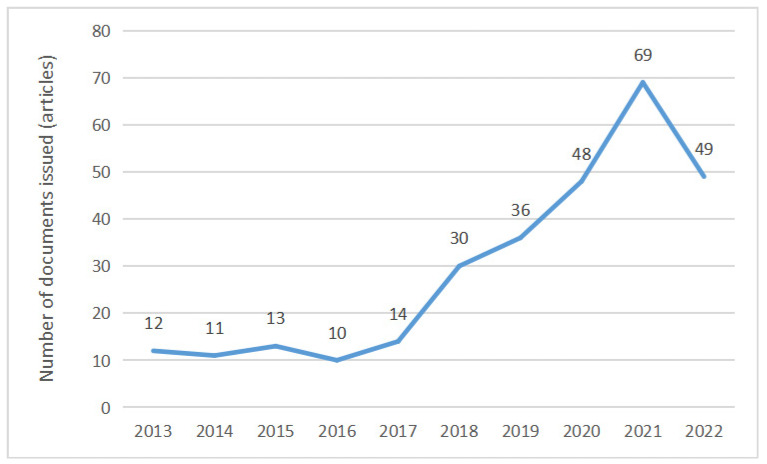
General trend in the literature on rural human settlement environments.

Figure 1 shows the trend in the research literature on rural human settlements in China in the past decade. It can be seen from Figure 1 that, overall, the literature on rural human settlements shows a linear growth trend. According to the growth rate and changes, the literature growth in the past ten years can be divided into three stages. First, from 2013 to 2016, the number of core papers published is relatively stable, with approximately 10 papers published annually. Second, from 2017 to 2021, the number of core papers published in this stage increased rapidly and the field was in a stage of rapid development. In 2017, the report of the 19th National Congress of the Communist Party of China formally proposed rural human settlement environment remediation action. In 2018, the General Office of the CPC Central Committee and the General Office of the State Council issued the Three-year Action Plan for the improvement of Rural Human Settlements (2018–2020). Therefore, the importance of the study of rural human settlement environments was enhanced in China academic circles. The third phase spans from 2021 to now. In 2021, the General Office of the CPC Central Committee and the General Office of the State Council issued the Five-Year Action Plan for the Improvement of Rural Human Settlement Environments (2021–2025), which indicates that the governance of rural human settlement environments has entered a new stage. According to the trend in publications and the current policy requirements, in the next few years, the number of people studying rural human settlement environments may continue to rise.

**Figure 2:**
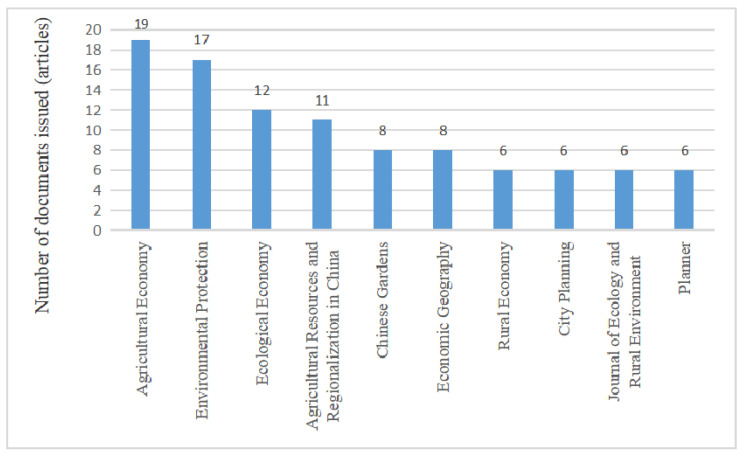
Distribution of literature sources of rural human settlement environment research.

The journal sources that published more than six articles in the past ten years were selected for inclusion in this paper (see Figure 2). According to the annual distribution of literature source journals, it can be seen that *Agricultural Economics* has published the most articles, with 19 articles in total. The second is *Environmental Protection*, with 17 articles. Most of them belong to the social humanities class of journals, indicating that, in the China academic community, the humanities attach more importance to the study of rural human settlement environments.

### 3.2. Authors of Research on Rural Human Settlement Environments

According to the data analysis of 292 studies by SATI, the total number of authors who have published work on rural human settlements in the past decade is 717, and the number of independent authors is approximately 612, with an average of 2.46 authors per paper. Table 1 offers a list of the authors who have published more papers in the field of rural human settlements (more than three papers). The author with the most papers is Li Bohua (16 papers). The second is Liu Peilin, with 10 papers.

**Figure 3:**
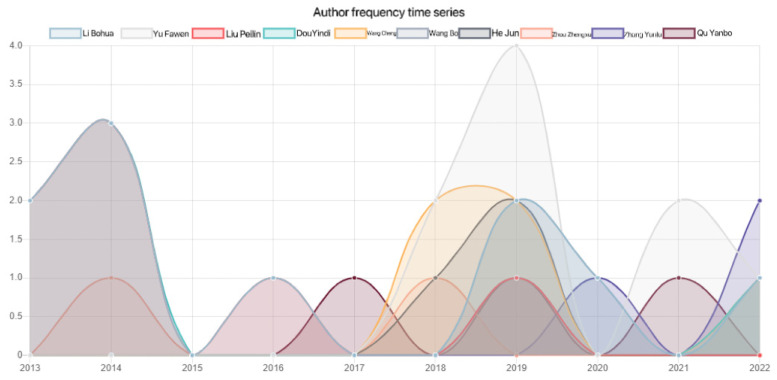
Author publication frequency in relation to rural human settlement environment research.

**Figure 4:**
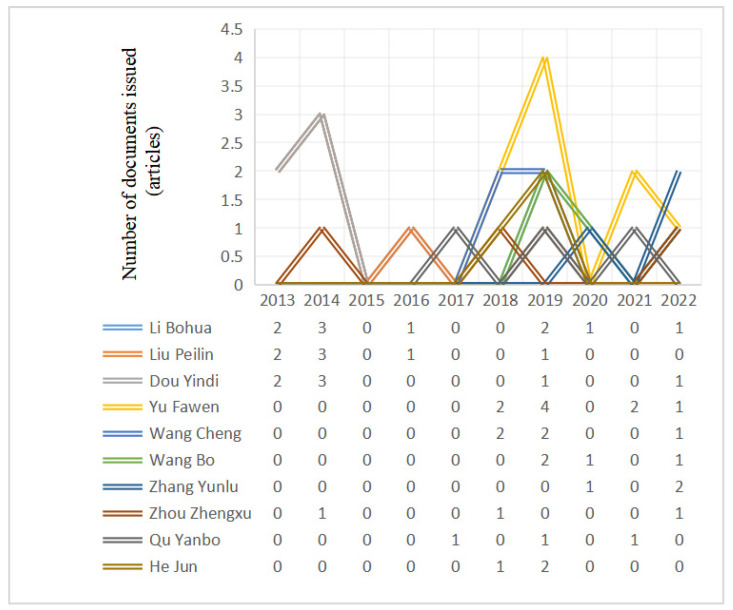
Author publication trends for rural human settlement environment research.

Using SATI, the publication frequency of the top 10 authors in the last 10 years was analyzed, and Figure 3 was obtained. In order to make the data clearer, Figure 4 was drawn according to the data obtained in Figure 3. It was found that Li Bohua was the most active author, with almost annual output from 2013 to 2022. Li Bohua has the longest research time, while Zhang Yunlu has the shortest research time. The first paper was published in 2020.

**Figure 5:**
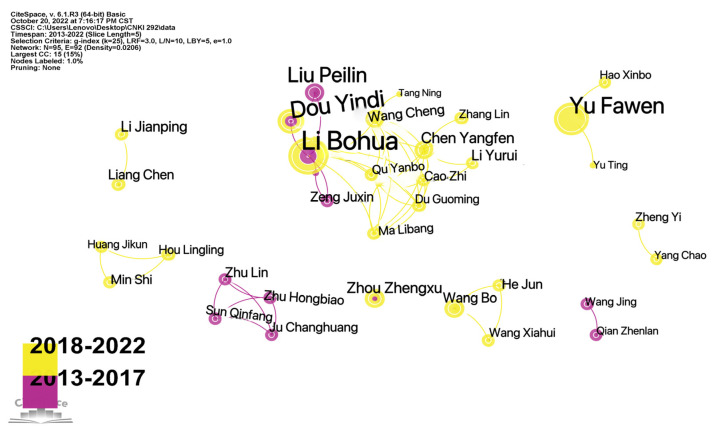
Knowledge map of the author cooperation network for rural human settlements research from 2013 to 2022.

In order to further understand the cooperation between authors, the authors were analyzed using CiteSpace V. The time slice was set to five years, and other settings were set to the default. Authors without networks were removed, and an author cooperation network diagram of rural human settlement environment research was obtained, as shown in Figure 5. In Figure 5, we can see eight groups of authors who cooperate with each other, represented by Li Bohua, Yu Fawen, Wang Bo, etc. Among them, the author cooperative group with Li Bohua at its core is the most extensive and the largest. This core group of authors has had an important influence on the development of the field. Chinese scholars have formed a core network, which indicates that the Chinese research on rural human settlement environments has gradually matured.

### 3.3. Research Institutions Engaging in Rural Human Settlements Research

According to the SATI analysis, from 2013 to 2022, a total of 494 institutions participated in related research, including 386 independent institutions, with an average of 1.69 publishing institutions per article. Table 2 shows the institutions that published more than four articles; of these, the Institute of Rural Development of the Chinese Academy of Social Sciences published the most, with a total of 10 core articles or more. The Institute of Geographic Sciences and Natural Resources Research of CAS and the Hunan Human Settlement and Environment Research Base both published six articles. As can be seen from Table 2, comprehensive research institutions in the field of agriculture and the ecological environment are the main driving forces in the study of rural human settlements. Among these institutions, Beijing, Hunan, and Hubei are the most representative, indicating that developed areas have certain advantages in the study of rural human settlements.

**Figure 6:**
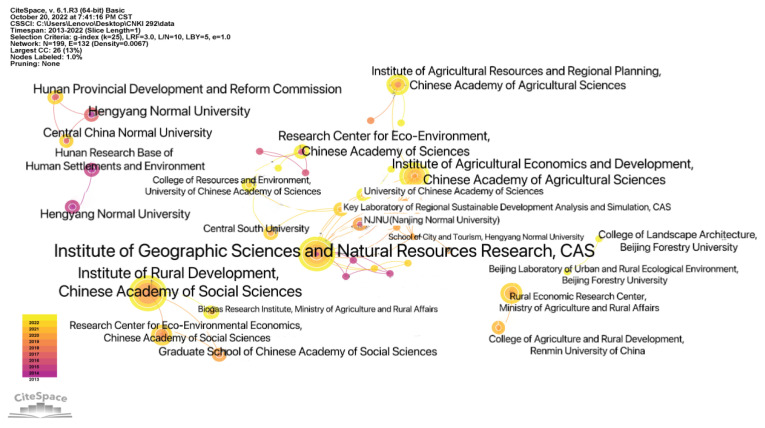
Knowledge map of the cooperative network of rural human settlement environment research institutions.

In order to further understand the cooperation among institutions, CiteSpace V was used to analyze the institutions, eliminate the institutions that have not formed connections, and obtain a knowledge map of the cooperation network of institutions researching rural human settlement environments; this map is shown in Figure 6. Figure 6 shows six groups of institutions forming cooperative networks, which are represented by the Institute of Rural Development, the Chinese Academy of Social Sciences, the Institute of Geographic Sciences and Natural Resources Research, the Chinese Academy of Sciences, the Research Center for Rural Economy, the Ministry of Agriculture and Rural Affairs, Hubei Key Laboratory of Geographical Process Analysis and Simulation, Central China Normal University, and the College of Landscape Architecture, Beijing Forestry University. These institutions also have a high number of publications; among them, the Institute of Geographic Sciences and Natural Resources Research of the Chinese Academy of Sciences has cooperated with the most institutions and is most closely connected with other institutions, indicating that this institute plays a leading role in cooperation between institutions researching rural human settlements.

### 3.4. Research Hotspots for Rural Human Settlement Environment Studies

SATI software (http://www.sationline.cn/#!/, Tianjin, China) was used to analyze the literature data, and 1407 keywords were found from 2013 to 2022, among which 895 were independent keywords, with an average of 4.82 keywords per article. Table 3 shows 20 key words with a frequency of ≥6. Among them, the frequency of “rural human settlement environment” is as high as 108, accounting for 6.18% of the total keywords, followed by the frequency of “human settlement environment” and “rural revitalization” with 89 and 63, respectively, indicating that the rural human settlement environment has an important position in the literature.

**Figure 7:**
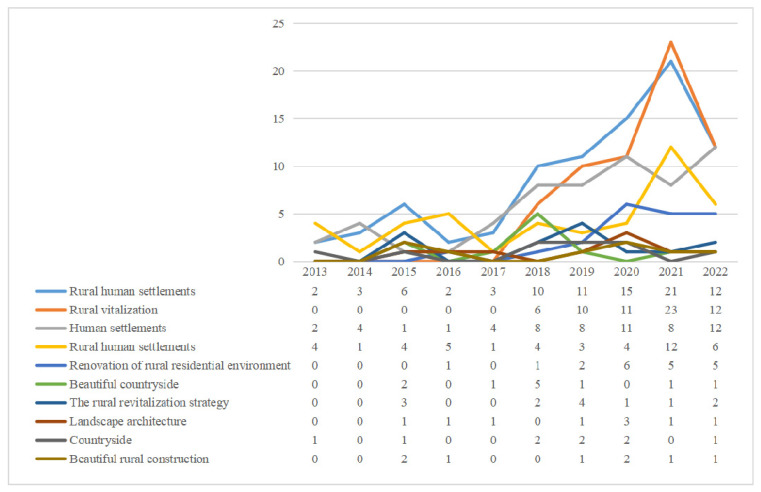
Keyword trend chart of rural human settlement environment research.

The top 10 words were plotted into the word frequency trend chart (Figure 7). The frequency of “rural human settlements” has increased rapidly since 2018 and is, so far, in the highest frequency position. In addition, it can be seen from the figure that “rural human settlement environment” and “rural human settlement environment” appear every year, and their frequency continues to rise with the appearance and growth of the word frequency of “rural revitalization” from 2018. This indicates that rural human settlements have received constant attention from the domestic academic community. The proposal and implementation of the rural revitalization strategy have further promoted the development of rural human settlements, and a good rural human settlements environment has become an important part of the realization of rural revitalization.

**Figure 8:**
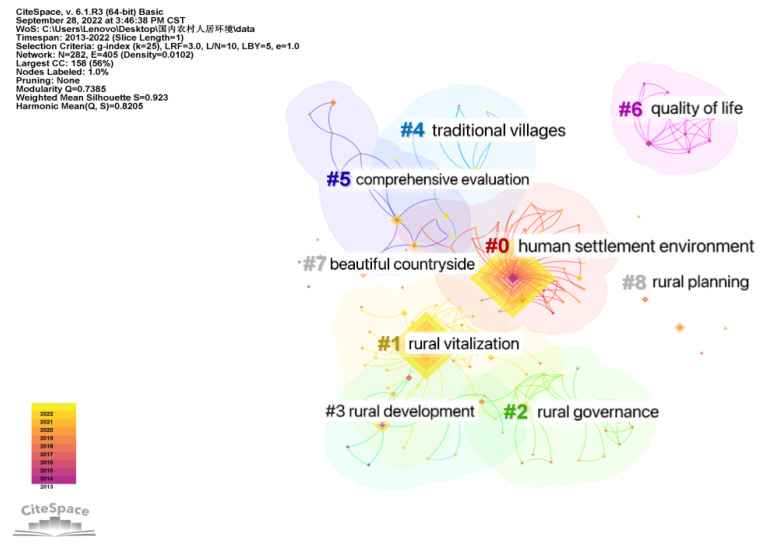
Keyword clustering of rural human settlement environment research.

Bibliographic information in a RefWorks format was imported into CiteSpace V software, and the type of keyword node was analyzed. The time slice was set to one year, and the other settings were set to default. After running, the LLR (Log Likelihood Ratio) algorithm was used to obtain the rural human settlements keyword clustering diagram (Figure 8). There are 282 nodes and 405 connections in the graph, the network density is 0.0102, the Q value is 0.7385, and the S value is 0.923, which indicates that the clustering structure is significant, and the clustering reliability is very high.

**Figure 9:**
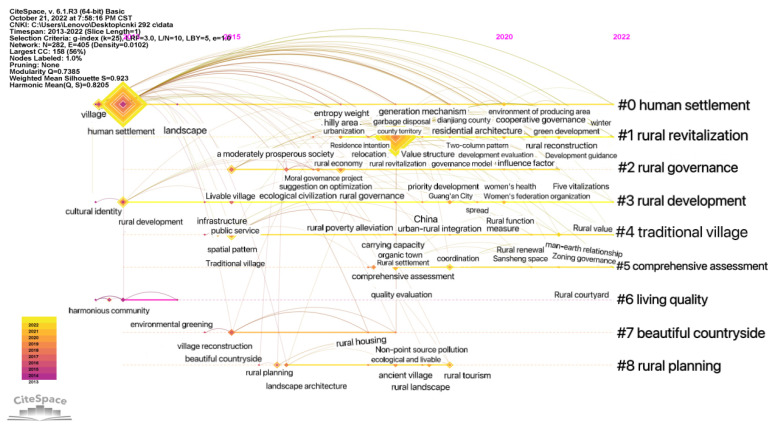
Timeline of keywords in rural human settlements research.

**Figure 10:**
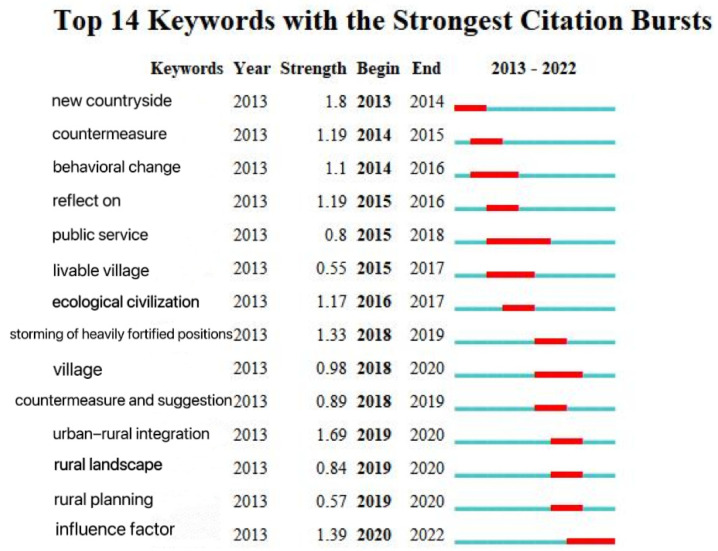
Prominence of keywords in rural human settlement environment research.

The smaller the number in the clustering label, the more keywords it contains. Taking the first three significant clusters as an example (Figure 9), the clustering of literature on human settlements first appeared in Qu Hanfei in 2013. In the Current Situation and Improvement Strategy of Rural Human Settlements [4], the clustering results began to increase from 2013, and, as time passed, the attention paid to this cluster has been maintained at a high level. In the keyword prominence view (Figure 10), “new rural areas” in 2013 and “livable rural areas” in 2015–2017 showed important achievements, indicating a major turning point in the field. For example, CAI Jin’s 2013 article “The construction of new rural communities” played a significant role in improving rural living environments [5]. In 2016, Xu Wenhui proposed the planning and construction strategy of “suitable for living, working, traveling and literature”, analyzed the weight of “four suitable” indicators, and proposed the construction content and strategy [6]. The literature in the rural revitalization cluster first appeared in 2018, with Zhang Weimin’s “Improvement of Rural Living Environment Focuses on overall planning” [7], Yu Fawen’s “Thinking on Countermeasures for Green Rural Development in the New Era” [8], and Shi Lei and Zheng Shan’s “Construction Mechanism of Rural Human Settlements under the Strategy of “Rural Revitalization”: Practical Experience and Inspiration of the European Union” [9]. In the same year, the clustering results increased, and the research heat kept rising. From 2020, the number of results increased rapidly. The rural governance literature cluster first appeared in 2018 with Liu Youtian’s “Research on Countermeasures to Promote the Pilot work of Rural Community Construction” [10]. Since 2018, the clustering results have increased. As shown in Figure 10, the keywords led by rural areas, critical battles, and countermeasures and suggestions reached an obvious turning point from 2018–2020, and important research results appeared. For example, Zhang Hongyu emphasized the importance of prioritizing agricultural and rural development in realizing rural revitalization [11]. Elsewhere, Wu Huifang and Wang Yuxia discuss how to better leverage the role of women and women’s organizations in rural development [12].

According to the above graphic analysis, the research themes and trends related to rural human settlements in CNKI can be divided into the following areas.

The first point relates to rural revitalization and rural human settlement environment research. At present, most Chinese research on rural human settlements is related to the development of rural revitalization. Based on the background of rural revitalization, Liu Chunxia’s paper analyzes the current status of rural residential environment improvement in Henan Province, and puts forward countermeasures, such as developing rural industries, unified village planning, and the intensive use of public funds [13]. Li Bohua also focuses on rural revitalization [14]. Li et al. studied the path of transformation and development of human settlements in traditional villages and analyzed the connection between the transformation and development of human settlements in traditional villages and rural revitalization. Huang Fubin analyzed the policy effects of photovoltaic poverty alleviation on promoting rural revitalization in relation to village governance, industrial development, and the living environment, and argued that the policy effects of photovoltaic poverty alleviation on rural revitalization are mainly reflected in the promotion of industrial development and an improved living environment, living conditions, and infrastructure conditions [15]. From the perspective of ecological value, the improvement of the living environment and other fields, Yan Qiying’s paper analyzes the constraints placed upon rural ecological vitalization and puts forward the appropriate measures for rural ecological vitalization [16]. The construction of rural human settlement environments and the realization of rural revitalization are interrelated and inseparable. The effective governance of rural human settlement environments will promote the development of rural revitalization, and the implementation of rural revitalization strategies is conducive to improving the governance of rural human settlement environments.

The second point pertains to research on the residential environment. Residential environment research is the focus of this area of rural residential environment research, and mainly considers water and sanitation improvement, garbage treatment, sewage treatment, and the construction of public service facilities. It advocates the construction of a new countryside and a livable countryside. However, domestic research on the living environment focuses more on the improvement of the natural environment, and less on the study of the human and cultural environment in relation to the rural living environment. Zhou Chuanbin designed an ecological engineering model for a rural courtyard, with “planting—breeding—toilet—cleaning—water cellar” as the main line, demonstrating a micro-ecological cycle, with the production and life integration of a compound ecological courtyard [17]. Ma Junqi indicates that the overall quality of rural human settlements is high, but there are significant differences in all aspects [18]. There have been obvious achievements in the ecological environment and infrastructure; meanwhile, there are still some weaknesses in the cultural environment, public services, and housing conditions. The governance effect of rural human settlements in eastern China is obviously better than that in central and western China. Plain terrain, reduced labor outflow, collective economic development, the management experience of village cadres, the number of village Party members, and donations of returning residents can significantly improve the quality of rural human settlements. To increase the fiscal tilt to the central and western regions, channels of talent flow should be improved, the collective economy strengthened, new rural sages actively cultivated, and the self-governance mechanism improved, so as to further improve the efficacy of rural environmental governance.

The third area pertains to residential space research. China is a vast country. Due to the varying climate, terrain, and other natural environmental features, as well as traditional national culture and social and economic conditions, there are regional differences in residential spaces. Lin Chuanhong analyzes the spatial patterns of rural human settlements in Anhui Province from three perspectives: production space, living space, and ecological space [19]. From the perspective of urban and rural integration, Liu Min advocates that the governance of rural human settlements should be coordinated from the perspectives of governance subjects, governance methods, and governance models, while paying attention to integrity [20]. An Wenyu et al. summarized the mapping co-occurrence relationship between ecological restoration in China’s territorial space and rural revitalization and explored the implementation paths and technological integration model of the ecological restoration of rural territorial space from the perspective of rural livelihood spaces [21]. By analyzing the picture of rural human settlements in the ideal state of the literati and officials depicted by painter Wang Ximeng of the Northern Song Dynasty, Wang Lin et al. explored the landscape system and characteristics of rural settlements in relation to the four aspects of overall layout, architecture, trees, and activities. They found that its formation motivations were the environmental view, social background, aesthetic thought, and life attitude, which provided references for the construction of rural human settlements at present [22]. Li Bohua contends that the transformation and upgrading of human settlements in traditional villages depend on the conversion of material and energy between the inside and outside of the system [23]. Combining the basic methods of ecology, geography, and environmental science, the transformation and development stages of traditional village human settlements are divided into four stages—invasion, competition, response, and regulation—and conceptual models of the driving mechanisms of different stages are constructed.

## 4. The Research Status and Differentiation Analysis of Rural Human Settlement Environments in WOS

As shown above, a knowledge graph analysis was undertaken for the research of rural human settlements in CNKI. Then, statistics and trend analysis were conducted for the research of rural human settlements with the data of literature included in WOS, so as to understand the overall and directional nature of international research in this field. This aspect of the study aimed to generate follow-up research on rural human settlements.

### 4.1. The Number of Publications on Rural Human Settlement Environments

**Figure 11:**
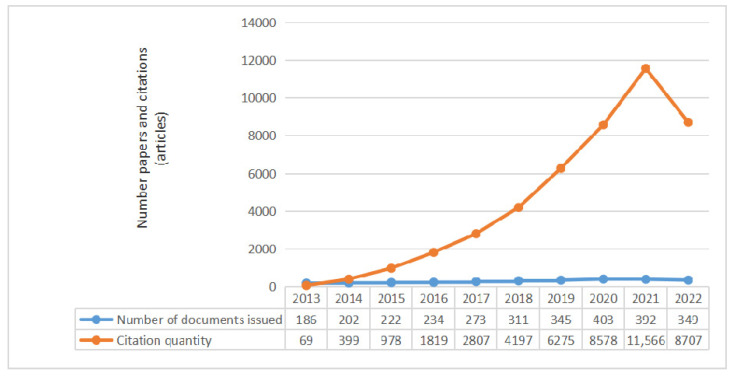
Trends in publications and citations in rural human settlements research.

As can be seen from Figure 11, the number of published documents is on the rise. Since 2022 had not yet ended at the time this manuscript was prepared, the data for 2022 are incomplete. According to the overall trend, the number of published documents and citations of historic policy in 2022 is predicted to exceed that in 2021. According to Figure 11, the rural human settlement environment was not paid much scholarly attention before 2015, with slow growth and a low number of citations. After 2015, the number of citations exceeded 1000, and from 2021 onwards, the number of citations exceeded 10,000. Excluding self-citation, each paper was cited 15.18 times, with a total of 45,408 citations. This indicates that, from 2013 to 2022, English research achievements on the rural human settlement environment gradually became abundant, and the research prospects of rural human settlement environments were broadened.

**Figure 12:**
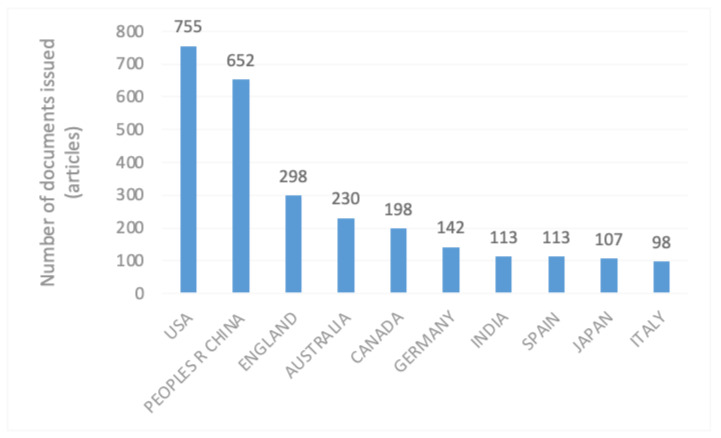
National distribution statistics of rural human settlement environment research.

In terms of the countries that have published papers on rural human settlements, this paper selects the top 10 countries according to the number of published papers (Figure 12). It can be seen from the figure that 7 of the top 10 countries are European and American countries, and the remaining 3 are Asian countries. Among them, the United States published the highest number of articles, and the first scholar to pay attention to the rural living environment was an American, the famous sociologist Peter Berg [24]. This shows that the study of rural human settlements is most mature in the United States and has accumulated richly in this country. China ranked second only to the US in terms of the number of published papers. This indicates that China is also making efforts to strengthen the construction of rural human settlements. It has conducted in-depth explorations of rural human settlements; the research level has continuously improved, and good results have been achieved.

**Figure 13:**
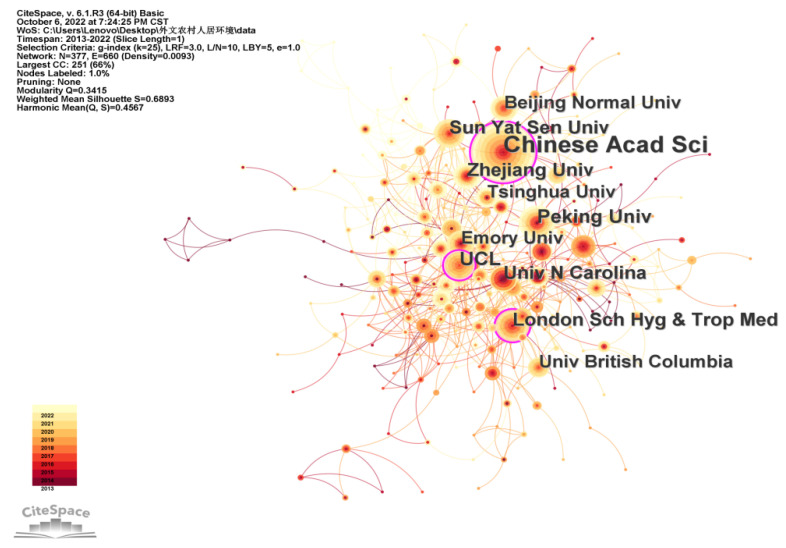
Institutional cooperation network of rural human settlement environment research.

According to the analysis of the network of cooperative institutions that was conducted using CiteSpace V software (see Figure 13), there are 377 nodes in the network, among which the highest intermediary centrality is China Science, which plays an important role as a bridge among cooperative institutions. In addition, University College London and the London School of Hygiene and Tropical Medicine have a high degree of intermediary centrality, indicating that they also play an intermediary role among the cooperative institutions. As can be seen from the figure, foreign cooperative institutions conducting rural human settlement environment research are closely connected, and a relatively complete cooperative network has been formed. It can also be seen from the figure that famous domestic universities, such as Peking University, Tsinghua University, Zhejiang University, and other famous foreign universities have the same close cooperation and exchanges. The exchange and cooperation between domestic and foreign research institutions is a favorable factor for promoting the further development of rural human settlement environment research.

The top 10 disciplines of English research on rural human settlements were selected (see Table 4), and the top 3 publication areas were ecological environmental science (909 papers), public environmental occupational health (661), and science and technology (315), indicating that WOS research on rural human settlements was dominated by the natural sciences. Social sciences, including psychology, public administration, and urban studies, accounted for only 9.729% of the literature. This shows that, in place of theoretical research, practice and technical application are the most important factors.

### 4.2. Authors of Studies on Rural Human Settlements

According to the SATI analysis, the number of total authors and independent authors in the study of rural human settlements in the past decade is 15,290 and 13,662, respectively, with an average of 5.12 authors per article. Table 5 shows the authors who published more than 14 articles. It can be seen from the table that Wang Y. published the most articles (43), followed by Zhang Y. with 42. Among the authors with more than ten papers, most are Chinese authors, which indicates that the Chinese research level is relatively high.

**Figure 14:**
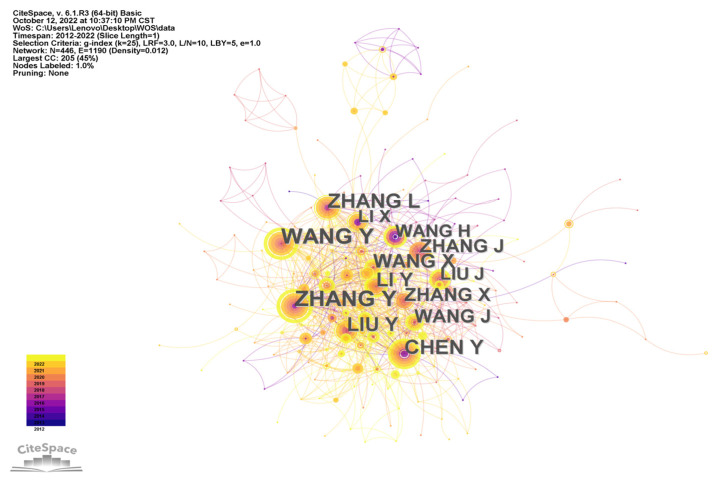
Author cooperative network for research on rural human settlements.

Through an analysis of the author cooperation network, conducted using CiteSpace, Figure 14 was obtained. According to Figure 14, a relatively complete cooperation network has been formed among authors of WOS studies on rural human settlements in the last ten years. Among them, Zhang Y. has the highest intermediation centrality and has the closest connection with other authors. There is also a close relationship between Chinese authors and other nations’ authors, which provides good conditions for the development of international research on rural human settlement environments.

### 4.3. Status of Important Nodes in the Literature on Rural Human Settlement Environments

**Figure 15:**
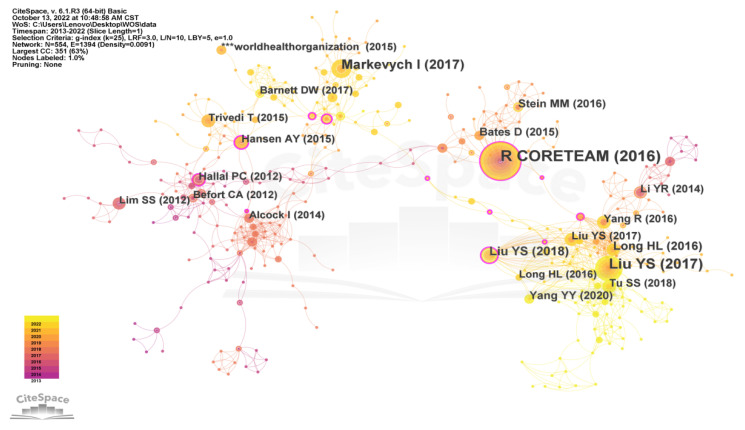
The citation network of rural human settlement environment research.

The type of reference nodes in the literature data was analyzed using CiteSpace V, and the time slice was set as one year with a time span of 2013–2022. Other settings were set to the default. Figure 15 and Table 6 were obtained from this analysis. As can be seen from the table, among the 10 key literature nodes on rural human settlements, 6 were authored by Chinese authors, indicating that Chinese scholars pay a good deal of attention to the relevant international research on rural human settlements. Among the 10 key literature nodes, Bates, D. published the earliest papers The structure of Linear Mixed Effects Models, the steps for evaluating contour bias and REML criteria, and the structure of the class or type representing such models were described in the Fitting Linear Mixed Effects Models using lme4, which allows users who wish to write functions to fit a particular linear mixed effects model to specialize these structures [25]. Liu Y.S.’s article titled “Revitalize the world’s countryside” argues that rural revitalization is needed to cope with global urbanization [26]. Four suggestions are put forward. First, the government should promote rural urbanization at the same time. Small cities and towns can act as bridges between remote villages and cities. Second, bottom-up initiatives should be encouraged. A visionary and professional group of local stakeholders (including entrepreneurs, workshop owners, and farmers) is needed to drive rural development. Third, some uninhabitable villages must be relocated. Fourth, a scientific plan to guide the rural revitalization process is highly necessary. In addition, in her *Introduction to land use and rural sustainability in China*, Liu Y.S. offered an examination of the impact of shifts in human socio-economic activities on changes in land use and related policy making from both Chinese and global perspectives [27]. Conceptual theory and empirical research are provided for research topics such as urbanization and arable land protection, rural transformation and reconstruction, and rural–urban interaction in changing societies. In “Exploring pathways linking greenspace to health: Theoretical and methodological guidance links green space to health”, Markevych, I. confirms the beneficial effects of green spaces on health and emphasizes three general functions and interrelationships of green space: harm reduction (e.g., reduced exposure to air pollution, noise, and heat), resilience (e.g., attention recovery and recovery from physiological stress), and building capacity (e.g., encouraging physical activity and promoting social cohesion) [28]. Thus, the relationship between human settlements and health is an important theme in WOS studies of rural human settlements.

### 4.4. Rural Residential Environment Research Hotspots

A total of 11,635 keywords and 6968 independent keywords were retrieved through WOS, with an average of 3.89 keywords per article. According to the word frequency list of CiteSpace V, the top 20 high-frequency keywords were selected (see Table 7). Among these words, “health” is the most frequent, followed by “environment”, representing the research focus of foreign rural human settlement studies. Foreign studies on rural human settlements pay more attention to human physical and mental health and urban environments, which are bound up with physical activity, mental health, urban environments, urbanization, and community.

**Figure 16:**
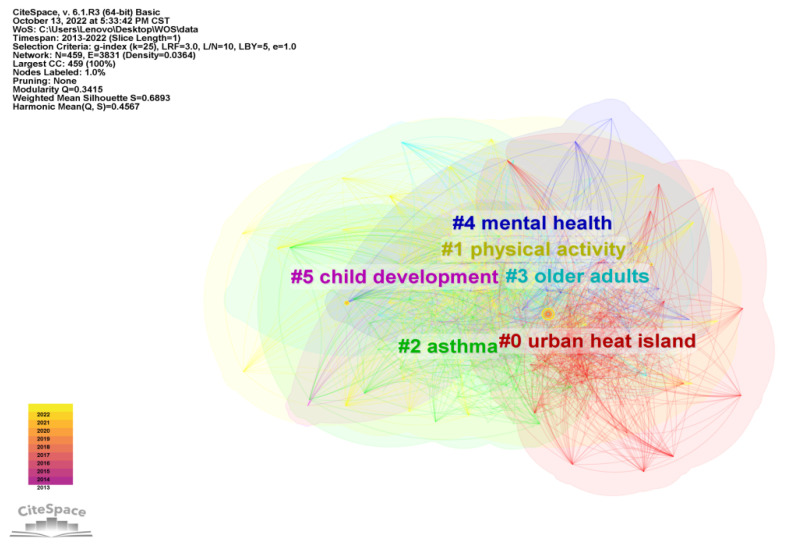
Keywords clustering in rural human settlements research.

The WOS bibliographic information was imported into CiteSpace V software, the type of Keyword node was analyzed, the time slice was set to one year, and the keyword clustering view was obtained after running the software (Figure 16). There was a total of 459 nodes and 3831 connections, the network density was 0.0364, the Q value was 0.3415, and the S value was 0.6893. Q > 0.3 indicates a significant clustering structure, S > 0.5 indicates reasonable clustering.

**Figure 17:**
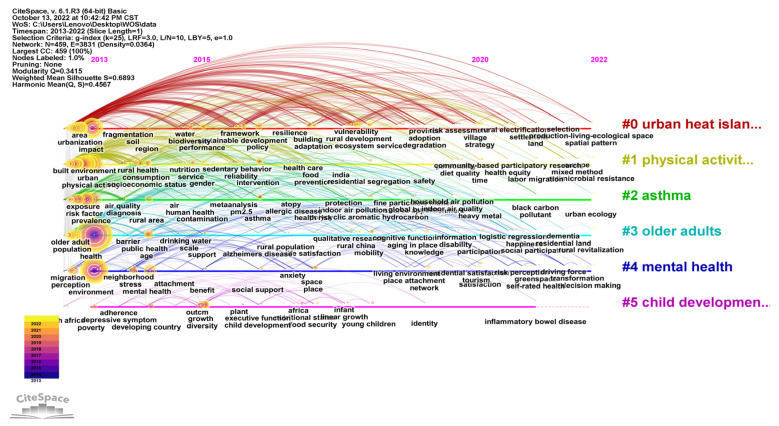
Timeline of keywords in rural human settlements research.

**Figure 18:**
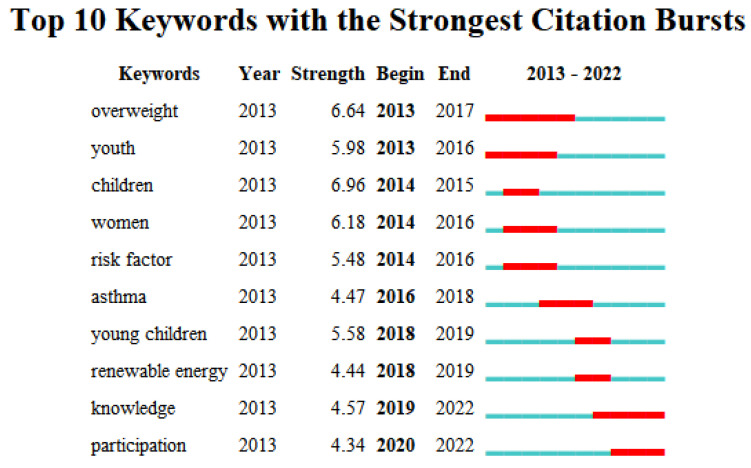
Keywords of rural human settlement environment research.

The keyword clustering was converted into a time graph (Figure 17), showing five clusters in total. They are “urban heat island”, “physical activity”, “asthma”, “older adults”, and “child development”. The keywords under the first cluster first appeared in 2013 with Yoon, J., et al., “Testing the Effects of Constraints on Climate Change-Friendly Behavior among Groups of Australian Residents” [29]. The authors suggest that general attitudes towards climate change, subjective norms, and perceived behavioral controls can predict expected and reported behavior, and that attitudes can negatively affect the constraints of adopting ERBs. Moreover, these authors showed that the most important predictor of intent is perceived behavioral control. Published in the same year, Radford, K.’s “Changes in the value of ecosystem services along a rural–urban gradient: A case study of Greater Manchester, UK” focused on gradient changes in the value of nine ecosystem services (aesthetic, spiritual, recreational, water flow regulation, carbon uptake, climate change adaptation, pollination, biodiversity potential, and noise attenuation) across four urbanization categories (urban, suburban, urban fringe, and rural) [30]. Keywords under the second cluster appeared in 2013 with Beyer, K.’s “Characteristics of the Residential Neighborhood Environment Differentiate Intimate Partner Femicide in Urban Versus In Rural Settings”. This paper studied the role of neighborhood factors in distinguishing urban and rural IPF (intimate partner femicide) in Wisconsin, USA [31]. The results showed that the neighborhood environment plays different roles in affecting the risk of intimate violence in rural areas. Keywords under the third cluster first appeared in 2013 with Johnson, L., “The impact of GPX1 on the association of groundwater selenium and depression: a project Frontier study”, which explores the association between groundwater selenium levels and reduced depressive symptoms and identifies gene–environment interactions between selenium exposure and GPX1 (glutathione peroxidase 1) polymorphisms [32]. In combination with the list of emergent words (Figure 18), “overweight” has the highest intensity and attention continues to rise from 2013 to 2017. Youth, children, women, and young children have also attracted people’s attention in the past decade. This indicates that, in the last decade, WOS has attached great importance to the study of residential subjects in rural human settlement environments.

According to the above chart analysis, the themes and trends in rural human settlements research in WOS can be summarized as follows.

The first area pertains to residential environment research. English literature mainly discusses the impact of living environments on human health, in relation to asthma, depression, obesity, and other diseases, as well as the relationship between mental health and neighborhood relations. Studies have shown that the degree of exposure to natural environments affects mental health. Beyer, K.’s article examining the relationship between green spaces and mental health and the role of green space in non-urban settings suggests that “greening” may be a potential strategy for improving populations’ mental health in the United States [33]. Engemann, K. investigates the potential link between green space and mental health in the Danish population; the results suggest that access to green space during childhood is associated with better mental health and contributes to better integration of the natural environment into urban planning and children’s lives [34]. Vernon-Feagans, L. suggests that, although poverty and its associated risks and negative parenting styles are associated with poorer EF (early executive function) and behavioral regulation, chaotic home environments may also play a role in early EF and later behavioral regulation at school age [35]. Rautio, N. found that poor housing or built environments, including poor housing quality, inadequate functioning, a lack of green space, noise, and air pollution, are associated with depressed moods [36]. Kegler, M. examined the effects of rural family and community environments on healthy eating, physical activity, and body weight, and found that individual factors and the family environment have a direct influence on eating behaviors, while the neighborhood environment has an indirect influence [37]. Anderson, T. also demonstrated clear differences in health between rural and non-rural residents [38].

The study of residential subjects is another key area. In WOS, scholars have carried out classification research on the main part of residence when studying the rural residential environment, especially the elderly, women, children, and young people. The most important active groups in rural human settlements are the elderly, women, children, and other vulnerable groups. According to Burholt, V., the rural environment intensifies the difficulty of social participation and access to social resources, and it enhances the loneliness of the elderly in their later years [39]. In addition, Fan, J. found that the aging of China’s rural population increased rural household carbon emissions [40]. Singla, D.’s community-based study demonstrated a strong correlation between maternal mental health status and child development in rural areas [41]. At the same time, Odom, E. argues that there is a complex association between mothers’ work environments and developmental outcomes in African American children [42]. Mberu, B. conducted a comparative analysis of the health of slum, non-slum, and all urban and rural populations in Bangladesh, Kenya, Egypt, and India [43]. Focusing on comparisons of child health, maternal health, reproductive health, access to health services, and HIV, indicators of mortality and morbidity were found to be worse in slums than elsewhere, but indicators of access to care and coverage of health services were better in slums than in rural communities. Null, C. confirmed the close links between water quality, sanitation, growth, and diarrhea in children in rural areas, with malnutrition and exposure to fecal contamination associated with diarrhea and slowed growth, both of which can have long-term effects on children’s health [44].

## 5. Research Results

Based on the literature analysis conducted using SATI4.0, Note Express, and CiteSpace V, this paper drew a knowledge map of rural human settlements research from 2013 to 2022. We summarized and compared the number of papers, authors, research institutions, subject areas, research hotspots and trends, as well as important literature on rural human settlements in CNKI and WOS. The main conclusions are as follows:

### 5.1. The Number of Published Articles Showed an Upward Trend

In terms of the number of published papers, both CNKI and WOS have shown a rising trend in the number of papers published on rural human settlements research. Both of them attach great importance to rural human settlements research, and their research prospects are broad.

### 5.2. There Are Differences in Collaborative Studies

In terms of research authors and institutions, compared with WOS, CNKI lacks communication and cooperation between research authors and research institutions, and has not yet formed a complete and close cooperation network. However, Chinese research authors and research institutions have close cooperation with other countries’ research authors and research institutions internationally, and they play an important role in these networks. The core group of authors and cooperating institutions in international research on rural human settlement environment has formed a relatively complete cooperative network.

### 5.3. Higher Degree of Integration of Rural Human Settlements Research Disciplines in WOS

In this study, we can find that the study of rural human settlements in WOS integrates geography, environmental science, planning, ecology, economics, management, history, pathology, and other disciplines, realizing interdisciplinary research. The research on rural human settlements in CNKI has also achieved interdisciplinary research, but the degree of interdisciplinary integration is not as high as that in WOS.

### 5.4. Different Research Hotspots and Trends

The research hotspots in CNKI are rural revitalization, living environment, and living space, and more of them are based on rural revitalization to promote rural revitalization and development. It focuses on the transformation of the living environment, including the transformation of water and toilets, garbage disposal, and sewage treatment, and the construction of public service facilities. The living spaces of different regions are mainly studied from the perspectives of production, life, ecology, and “living space”, especially in relation to changes in traditional settlements in China. Because China has a long history and culture, the change in settlements reflects the different choices living subjects make regarding their living environments in different periods [45]. Generally speaking, China pays more attention to the macro-level of the rural human settlement environment and living natural ecological environment and other hard environmental research, and research on urban edge residential subjects, social relations, individual needs, and other soft environmental research is relatively sparse. The research focus in WOS is the residential environment and the residential subject, and more attention is paid to the health topic of the residential subject, the impact of the residential environment on the physical and mental health of the residential subject, and the social behavior relationship between different residential subjects in the residential environment. [46]. Such studies include the neighborhood environment and family environment, as well as focusing on the role of elderly people, children, women, and so on in different environments by assessing their impact or the impact on the environment. In addition, in the study of international human settlement environments, the comparative study of urban and rural human settlement environments, urbanization, and the human settlement environment in urban fringe areas are other research focuses. In short, WOS focuses on the study of individual psychological needs at the micro level, and lacks the analysis of residential layout, settlement changes, and other issues.

## 6. Conclusions and Prospects

It can be seen from the above studies that the research on rural human settlements has broad prospects. In future research, Chinese scholars should connect with the hotspots of international rural human settlements research, set out the national conditions, combine the actual situations of different regions and types of rural areas in China, and establish a system theory and policy suitable for the development of rural human settlements in China. Scholars should aim to strengthen the research on the subject of rural human settlements, social relations, individual needs, and the impact of urbanization on rural human settlements from a micro perspective and continue to conduct in-depth research on the human environment, housing structures, infrastructure, and urban–rural integration. We will continue to learn from countries with valuable practical experience and provide a meaningful reference for effectively promoting the integrated development of urban and rural areas in China, promoting rural revitalization and development and social equity.

## Figures and Tables

**Table 1:** Statistics on the number of papers published by certain authors on rural human settlements from 2013 to 2022.

No.	Author	Number of Publications (Articles)	Serial Number	Author	Number of Publications (Articles)
1	Li Bohua	16	10	Li Yurui	3
2	Liu Peilin	10	11	Zhang Yunlu	3
3	Dou Yindi	9	12	He Jun	3
4	Yu Fawen	9	13	Zhou Zhengxu	3
5	Zeng Juxin	8	14	Qu Yanbo	3
6	Wang Cheng	5	15	Yang Chao	3
7	Wang Xiahui	4	16	Zhu Lin	3
8	Wang Bo	4	17	Yang Qingqing	3
9	Li Bingdi	4	18	Chen Yangfen	3

**Table 2:** Statistics on the distribution of research institutions’ publications on rural human settlements.

No.	Institution	Districts	Number of Publications (Articles)
1	Institute of Rural Development, Chinese Academy of Social Sciences	Beijing	10
2	Institute of Geographic Sciences and Natural Resources Research, CAS	Beijing	6
3	Hunan Human Settlements and Environment Research Base	Hunan	6
4	Institute of Agricultural Economics and Development, Chinese Academy of Agricultural Sciences	Beijing	5
5	College of Economics and Management, China Agricultural University	Beijing	4
6	Institute of Agricultural Resources and Regional Planning, Chinese Academy of Agricultural Sciences	Beijing	4
7	Research Center for Eco-Environmental Economics, Chinese Academy of Social Sciences	Beijing	4
8	Rural Economic Research Center, Ministry of Agriculture and Rural Affairs	Beijing	4
9	Institute of Environmental Protection Research and Monitoring, Ministry of Agriculture and Rural Affairs	Beijing	4
10	College of Landscape Architecture, Beijing Forestry University	Beijing	4
11	Hubei Key Laboratory of Geographical Process Analysis and Simulation, Central China Normal University	Hubei Province	4
12	Department of Resources, Environment and Tourism Management, Hengyang Normal University	Hunan	4

**Table 3:** Statistics of high-frequency keywords in rural human settlement environment research.

No.	Keywords	Word Frequency (Order)	Serial Number	Keywords	Word Frequency (Order)
1	Rural living environment	108	11	Landscape architecture	9
2	Living environment	89	12	Beautiful countryside construction	8
3	Rural revitalization	63	13	Quality evaluation	8
4	Rural living environment	53	14	Influencing factors	8
5	Improvement of rural living environment	21	15	Traditional village	7
6	Rural	16	16	Rural governance	7
7	New countryside construction	12	17	Rural tourism	7
8	Rural revitalization strategy	11	18	Countermeasures	6
9	New countryside	11	19	Human settlement environment remediation	6
10	Beautiful country	11	20	Livable countryside	6

**Table 4:** Subject direction statistics of rural human settlement environment research.

Research Direction	Number of Publications (Articles)	Number of Articles (%)
Environmental Sciences and Ecology	909	30.391
Public Environmental Occupational Health	661	22.1
Science and Technology (Other Topics)	315	10.532
Engineering	163	5.45
Geography	119	3.979
Psychology	114	3.811
Health Care Science Services	93	3.109
Energy Fuels	91	3.042
Urban Studies	89	2.976
Public Administration	88	2.942

**Table 5:** Statistics for high-frequency authors of rural human settlements research.

No.	Author	Number of Publications (Articles)	Serial Number	Author	Number of Publications (Articles)
1	Wang Y.	43	11	Li X.	19
2	Zhang Y.	42	12	Liu J.	18
3	Chen Y.	31	13	Wang H.	18
4	Zhang L.	30	14	Chen H.	17
5	Li Y.	27	15	Li J.	17
6	Liu Y.	25	16	Wang Z.	16
7	Wang X.	24	17	Li Z.	16
8	Wang J.	22	18	Zhang Z.	15
9	Zhang X.	22	19	Wu Y.	14
10	Zhang J.	22			

**Table 6:** Key literature nodes of rural human settlement environment research.

No.	Author	Literature	Year	Number of Co-Citations
1	R Coreteam	R Lang Env Stat Comp	2016	30
2	Liu YS	Revitalize the world’s countryside.	2017	22
3	Markevych I	Exploring pathways linking greenspace to health: Theoretical and methodological guidance.	2017	17
4	Liu YS	Introduction to land use and rural sustainability in China.	2018	13
5	Long HL	The allocation and management of critical resources in rural China under restructuring: Problems and prospects.	2016	12
6	Bates D	Fitting Linear Mixed-Effects Models Using lme4	2015	10
7	Stein MM	Innate Immunity and Asthma Risk in Amish and Hutterite Farm Children.	2016	10
8	Yang YY	Coupling coordination analysis of rural production-living-ecological space in the Beijing-Tianjin-Hebei region	2020	10
9	Tu SS	Rural restructuring at village level under rapid urbanization in metropolitan suburbs of China and its implications innovations in land use policy.	2018	9
10	Yang R	Spatial distribution characteristics and optimized reconstruction analysis of China’s rural settlements during the Year The process of rapid urbanization.	2016	9

**Table 7:** High-frequency keywords statistics of rural human settlement environment research.

No.	Keywords	Word Frequency (Order)	Serial Number	Keywords	Word Frequency (Order)
1	health	311	11	exposure	110
2	environment	235	12	association	108
3	physical activity	171	13	community	108
4	impact	169	14	urbanization	101
5	prevalence	152	15	area	100
6	urban	151	16	population	98
7	built environment	132	17	mental health	94
8	risk	129	18	city	90
9	children	124	19	united states	82
10	risk factor	117	20	obesity	82

## Data Availability

The data used to support the findings of this study are available from the corresponding author upon request.
